# Probability-Based Early Warning for Seasonal Influenza in China: Model Development Study

**DOI:** 10.2196/73631

**Published:** 2025-08-06

**Authors:** Jinzhao Cui, Ting Zhang, Yifeng Shen, Xiaoli Wang, Liuyang Yang, Xuefeng Huang, Qiang Huang, Yu Yang, Weizhong Yang, Zhongjie Li

**Affiliations:** 1 School of Population Medicine and Public Health, Chinese Academy of Medical Sciences & Peking Union Medical College, No. 31, Beijigesantiao street, Dongcheng District, Beijing, 102206, China, 86 18612690539; 2 Shanghai Pudong New Area Center for Disease Control and Prevention (Shanghai Pudong New Area Health Supervision Institute), Shanghai, China; 3 Beijing Center for Disease Prevention and Control, Beijing, China; 4 School of Data Science, Fudan University, Shanghai, China

**Keywords:** seasonal influenza, machine learning, early warning, Dense Residual Network, public health surveillance, risk assessment

## Abstract

**Background:**

Seasonal influenza is a major global public health concern, leading to escalated morbidity and mortality rates. Traditional early warning models rely on binary (0/1) classification methods, which issue alerts only when predefined thresholds are crossed. However, these models exhibit inflexibility, often leading to false alarms or missed warnings and failing to provide granular risk assessments essential for decision-making. Therefore, we propose a probability-based early warning system using machine learning to mitigate these limitations and to offer continuous risk estimations of alerts (0‐1 variable) instead of rigid threshold-based alerts. Based on probabilistic prediction, public health experts can make more flexible decisions in combination with the actual situation, significantly reducing the uncertainty and pressure in the decision-making process and reducing the waste of public health resources and the risk of social panic.

**Objective:**

The main aim of this study is to devise an innovative approach for early warning systems focused on influenza-like cases. Therefore, a Dense Residual Network (Dense ResNet), a supervised deep learning model, was developed. The model’s training involved fitting the influenza-like illness positive rate, which enabled the early detection and warning of signals of changes occurring in the activity level of influenza-like cases. This departure from conventional methodologies underscores the transformative potential of machine learning, particularly in providing advanced capabilities for timely and proactive warnings in the context of influenza outbreaks.

**Methods:**

We developed a Dense ResNet machine learning model trained on influenza surveillance data from Northern and Southern China (2014‐2024). This model generates early warning signals 3, 5, and 7 days in advance, providing a probability-based risk assessment represented as a continuous variable ranging from 0 to 1, in contrast to the traditional binary (0/1) warning systems. We evaluated the performance of this model using area under the curve scores, accuracy, recall, and *F*_1_-scores, then compared it with support vector machine (SVM), random forests, XGBoost (Extreme Gradient Boosting), and LSTM (long short-term memory) models.

**Results:**

The Dense ResNet model demonstrated the best performance, characterized by 5-day lead warnings and a 50th percentile probability threshold, achieving area under the curve scores of 0.94 (Northern China) and 0.95 (Southern China). Relative to traditional models, probability-based warning signals improved early detection, reduced false alarms, and facilitated tiered public health responses.

**Conclusions:**

This study presented a novel probability-based machine learning model essential for early warning signals of influenza, demonstrating superior accuracy, flexibility, and practical applicability compared to other techniques. This approach enhances preparedness for influenza among the population and promotes the use of automated artificial intelligence–driven public health responses by replacing binary warnings with probability-driven risk assessments. Future research should integrate real-time surveillance data and dynamic transmission models to improve the precision of early warning.

## Introduction

Seasonal influenza is a contagious disease posing significant threats to human health globally, characterized by a steady increase in annual incidence. Currently, approximately 1 billion people worldwide are infected each year, and 290,000‐650,000 fatalities are due to influenza-related respiratory diseases [1,2].

This disease occurs in various seasons throughout the year, and its outbreak can be influenced by an environment conducive to viral survival, host immunity [3], patterns of physical contact with other people [4], or viral interference caused by other respiratory pathogens [5]. Therefore, a timely indication of an unusual increase in the prevalence of influenza is crucial; early detection and warning signals can mitigate the impact of this disease on public health, providing a foundation for the formulation of preventive measures and the timely allocation of medical resources. Previous studies have predominantly concentrated on predicting the trends of influenza outbreaks [6,7]. However, providing timely warning signals to the population is of greater practical significance for public health departments.

Various scenarios may trigger warning signals for influenza outbreaks, including surpassing historical baselines [8], excessively rapid increases in the prevalence trends, the discovery of new pathogens, increased disease severity, and insufficient medical resources [9]. This study concentrates on the warning scenario in which influenza transitions into an epidemic phase, characterized by an upward trend in the epidemic curve. In this scenario, the number of infected individuals increases, increasing demand for medical resources. As the prevalence rises, the disease burden intensifies steadily, necessitating specific personal protective or preventive measures to suppress the escalated outbreak and decelerate its transmission. Issuing a warning signal at this stage does not imply the emergence of a pandemic or a deviation from the usual trend; instead, it emphasizes the necessity for health departments and society to remain vigilant regarding the influenza epidemic.

The results of traditional early warning methods are categorized into 2 types: early warning signal and no early warning signal. An alarm will only be triggered when the expected threshold is exceeded. While these methods have been widely used in public health surveillance, they are constricted by several critical limitations [10,11]. For example, binary warning signals lack granularity, providing only rigid “yes” or “no” decisions rather than rendering a nuanced risk assessment. This often leads to the activation of false alarms or increases the likelihood of missed detections, reducing system reliability. Moreover, fixed-threshold models, such as the 40th percentile warning system, fail to distinguish between increasing and decreasing trends of the epidemic, which occasionally creates alerts even when cases are in decline. Finally, these models do not cater to tiered public health responses, making it difficult for decision-makers to prioritize interventions based on varying levels of outbreak risk [12]. Addressing these limitations requires a more flexible and adaptive approach, where warning signals are expressed as continuous probability values rather than binary classifications [11,12]. This study introduces a probability-based early warning model that enables tiered response strategies and artificial intelligence (AI)-driven automation, offering a significant improvement compared to traditional methods.

The rise of machine learning and AI methodologies provides a thrilling prospect for transforming early warning systems of influenza. Traditional warning models, such as the Moving Epidemic Method model [13], rely on historical data to predict the current prevalence of influenza. Machine learning methods, on the other hand, offer the potential to forecast warnings in advance. This shift toward advanced computational techniques not only improves the accuracy of predictions but also revolutionizes the ability of the population to anticipate and respond to public health threats.

Machine learning models such as the Self-Excitation Attention Residual Network have demonstrated the ability to dynamically adapt to evolving disease trends and changes in surveillance systems, enhancing the effectiveness of early warning systems [14]. These models can process complex, heterogeneous data from multiple sources, including cases of influenza-like illness (ILI), virological surveillance, climate and demography data, and resultant queries from search engines [15]. Consequently, they can identify intricate patterns and relationships that traditional models might miss, leading to more sensitive and accurate predictions.

Moreover, machine learning approaches have been shown to outperform traditional models with various traits such as sensitivity, specificity, and timeliness. For example, studies have found that machine learning models can issue warning signals before the onset of influenza outbreaks, exhibiting higher accuracy than threshold-based methods [16]. This enables public health officials to take preemptive measures, such as implementing vaccination campaigns or public advisories, to mitigate the impact of the disease.

Machine learning also demonstrates its potential for integrating various data sources and applying complex nonlinear models, such as long short-term memory (LSTM) or other neural networks, to the dataset, which can lead to higher prediction accuracy. These models can learn from genetic sequences and associated metadata to predict the antigenic properties of circulating influenza viruses, providing insights into the evolution of influenza and antigenic drift.

Integrating machine learning and AI into early warning systems of influenza represents a significant leap forward in our capacity to predict and prepare for influenza outbreaks. These advanced methodologies offer a more proactive, data-driven approach to public health surveillance, potentially saving lives and reducing the societal and economic burdens of influenza.

By constructing a Dense Residual Network (Dense ResNet) machine learning model to achieve probabilistic prediction of influenza outbreaks, it can effectively provide detailed probability information on the risk of an outbreak. Based on this probability prediction, public health experts can, in combination with their professional experience and the actual situation, make more flexible decisions on whether to issue a warning, avoiding the rigidity of the traditional binary decision-making model, significantly reducing the uncertainty and pressure in the decision-making process, and significantly reducing the waste of public health resources and the risk of social panic. The continuous probability model can provide more detailed epidemic warning information based on the risk level, supporting the implementation of graded intervention strategies by health departments, such as early deployment of medical resources and strengthening public health awareness campaigns when the probability is high, and internal monitoring and precise prevention measures when the probability is medium or low. This precise intervention effectively avoids unnecessary social panic and resource waste.

The main aim of this study is to devise an innovative approach for early warning systems focused on influenza-like cases. Therefore, a Dense ResNet, a supervised deep learning model, was developed. The model’s training involved fitting the influenza-like illness positive rate (ILI+), which enabled the early detection and warning of signals of changes occurring in the activity level of influenza-like cases. This departure from conventional methodologies underscores the transformative potential of machine learning, particularly in providing advanced capabilities for timely and proactive warnings in the context of influenza outbreaks.

## Methods

### Data Sources

**Figure 1.**
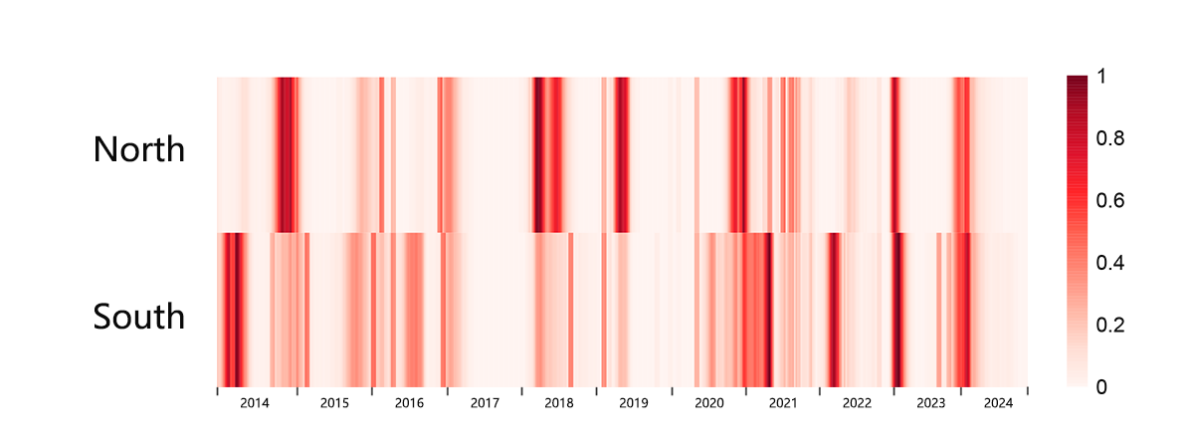
The influenza-like illness positive rate of Northern China (North) and Southern China (South).

The study population includes Northern China and Southern China. We derived proxy measures for the activity of the influenza virus in the communities, referred to as ILI+ rates, by multiplying the ILI percentage (ILI%) with the proportions of influenza-positive specimens [13] derived from the Chinese National Influenza Surveillance Network for the 2 regions. A total of 569 weekly reports were included in this study. The standardized ILI+ for each region was presented (Figure 1).

### Warning Definition and Labeling

**Figure 2.**
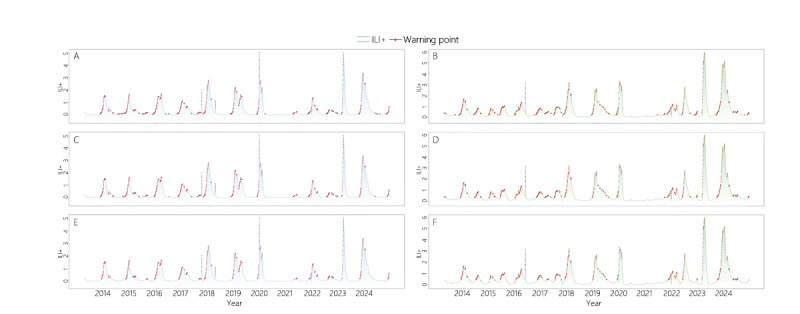
ILI+ warning diagram for Northern and Southern China under different quantile values. Panels A, C, and E refer to Northern China, panels B, D, and F refer to Southern China, panels A and B refer to quantile 0.4, panels C and D refer to quantile 0.5, and panels E and F refer to quantile 0.6. ILI+: influenza-like illness positive rate.

Throughout this study, the warning scenario was defined as an ascending trend during the influenza season. Following the conventional definition of the influenza season presented in previous studies [17], we designated a warning when ILI+ exceeds the 40th percentile, demonstrating an upward trend. The labeled outcomes are illustrated in Figure 2.

### Model Construction

**Figure 3.**
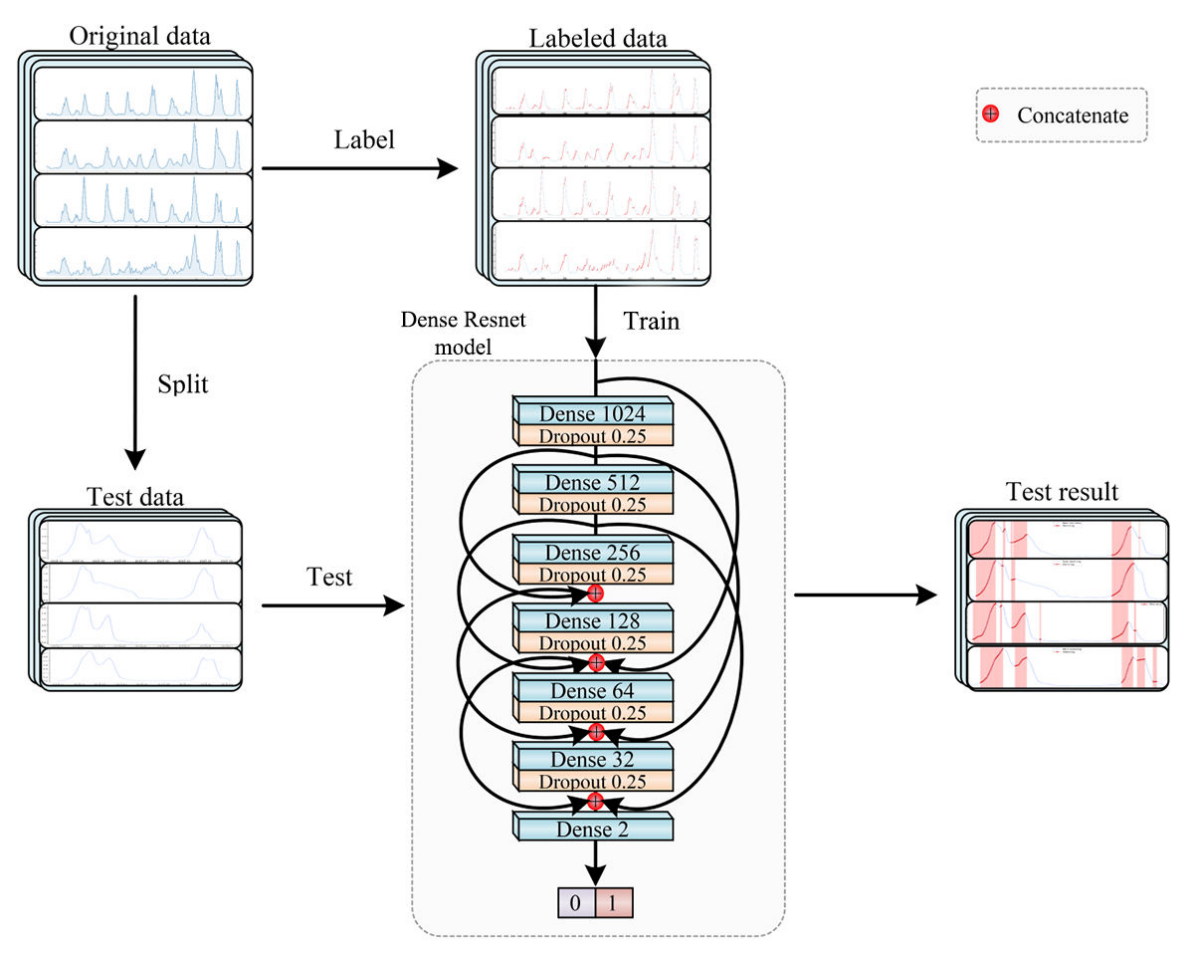
Structural diagram of the Dense Residual Network (Dense ResNet) model.

This study aimed to construct and train a model that could identify the intrinsic features of the input data and establish a mapping from ILI+ features to warning categories to accurately classify ILI+ for warning signals precisely. This was achieved by proposing an innovative deep learning model called Dense ResNet (Figure 3).

Dense layers and residual connection methods were used to design the Dense ResNet model to enhance the accuracy of the early warning function. Experimental results have demonstrated that this model could effectively map ILI+ features to the early warning category. The Dense ResNet model constructed in this study comprised 7 Dense layers, 6 Dropout layers, and 7 residual connections. From top to bottom, Dense layers of 1024, 512, 256, 128, 64, and 32 units were employed to extract data features from top to bottom. In addition, 6 layers with a dropout rate of 0.25 were applied after each Dense layer to prevent overfitting. Furthermore, 7 residual connections were added primarily to retain global information effectively while preventing gradient vanishing. The Sigmoid activation function was employed in the last fully connected layer to predict the probability of warning classes of 0 and 1. The predictive result was a probability value between 0 and 1 for each warning category.

In this study, Dense ResNet was selected over other deep learning models for time series early warning modeling of infectious diseases of the respiratory system, based on several key considerations. First, we used the ILI+ sequence as the feature for early warning judgments. Although it exhibited certain seasonality and trends, its variations were heavily influenced by external factors such as abrupt climate changes and public health interventions, leading to sudden shifts and nonstationarity. As a result, it does not adhere to consistent temporal evolution patterns. Traditional recurrent neural networks (LSTMs and Gated Recurrent Units), proficient in modeling strong sequential dependencies, are not entirely suitable for the ILI+ data used in this study. In contrast, Dense ResNet focuses on direct feature extraction, and by cumulatively integrating deep features, it is more adept at capturing the complex associations and nonlinear variation patterns present in the data, making it more compatible with the ILI+ features adopted in this research. Second, although models such as Transformers perform excellently in large-scale time series tasks, they have an escalated number of parameters that are highly prone to overfitting during the training process. This issue is particularly pronounced in this study due to the limited sample size of the ILI+ features, leading to poor generalization. In comparison, the proposed Dense ResNet structure is relatively simple; it mitigates the challenges of deep network training through effective residual connections, enabling stable training and superior performance on small to medium-sized datasets, offering higher practical application value.

In summary, considering the characteristics of the data, task requirements, model performance, training stability, and practical feasibility, this study ultimately adopted Dense ResNet for the early warning task instead of traditional recurrent neural network–based sequence models or large-scale Transformer architectures. This choice effectively balances the complex feature extraction capabilities, training stability, overfitting control, and deployment feasibility, providing strong technical support for achieving efficient and accurate early warning signals.

### Dataset Construction

**Figure 4.**
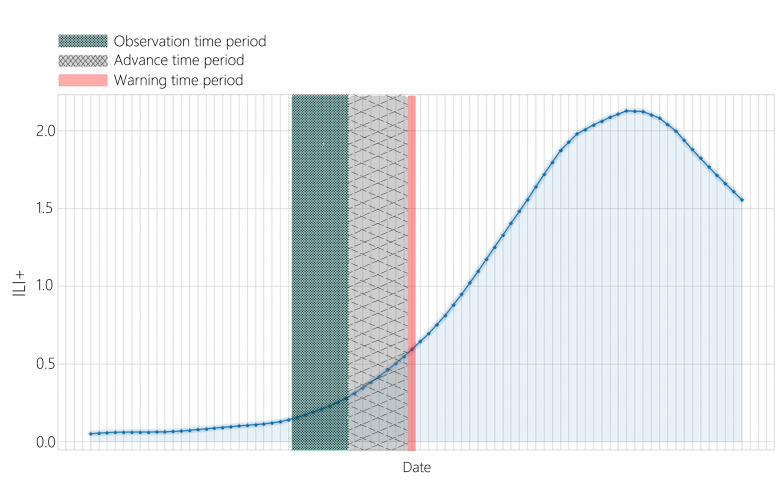
Schematic diagram of data construction. ILI+: influenza-like illness positive rate.

This study innovated the construction of the training dataset to activate early warning signals. Figure 2 illustrates the graphical refactoring of the dataset. We divided the data into 3 sections: observation time period, advance time period, and warning time point. We combine the features of the observation time period and the labels of warning time points to form a reconstructed dataset and train the model using this dataset to achieve early warning signals. For example, if a person needs to determine whether November 26, 2023, is a warning, you only need to observe the characteristic values from November 12, 2023, to November 18, 2023, will be observed (Figure 4).

In this study, for the choice of advanced time period, we adopted advanced time periods of 3, 5, and 7 days to select advanced periods, and the results were obtained.

### Dataset Segmentation

This section encompasses 3969 days spanning from December 30, 2013, to November 11, 2024, in Southern and Northern China, to construct training. Following the conventional division methods of deep learning, including training set, validation set, and test set, and considering the practical significance of ILI% prediction, the period from December 30, 2013, to October 31, 2022 was designated as the training set, while the period from November 1, 2022, to November 11, 2024 was assigned as the test set. The training set encompasses 9 ILI% activity peaks, while the test set contains 2 ILI% peaks.

### Model Training

In model training, we initialized the network weights using He normal initialization and trained the model using the Adam optimizer with default parameters (β₁=.900, β₂=.999). The initial learning rate was set to 0.001 with a batch size of 32. To further improve the model convergence, we employed a learning rate scheduler (ReduceLROnPlateau) that reduced the learning rate by 0.1 if the validation loss did not improve for 5 consecutive epochs. The binary cross-entropy loss function was used during training. The training was conducted for a maximum of 100 epochs. We adopted an early stopping strategy with a patience of 10 epochs based on the validation loss to prevent overfitting. The model development and experiments were implemented using the PyTorch framework (version 2.0.0; Meta AI). All the training, validation, and testing processes were performed on one NVIDIA RTX 4090 Graphics Processing Unit (GPU) with 24 GB of memory.

### Ethical Considerations

This study was approved by the Research Ethics Review Committee for Biomedical Research Involving Humans, Chinese Academy of Medical Sciences, and Peking Union Medical College (approval no. CAMS&PUMC-IEC-2025-010). The study did not involve any personally identifiable or sensitive health information. No compensation was provided to participants.

## Results

**Figure 5.**
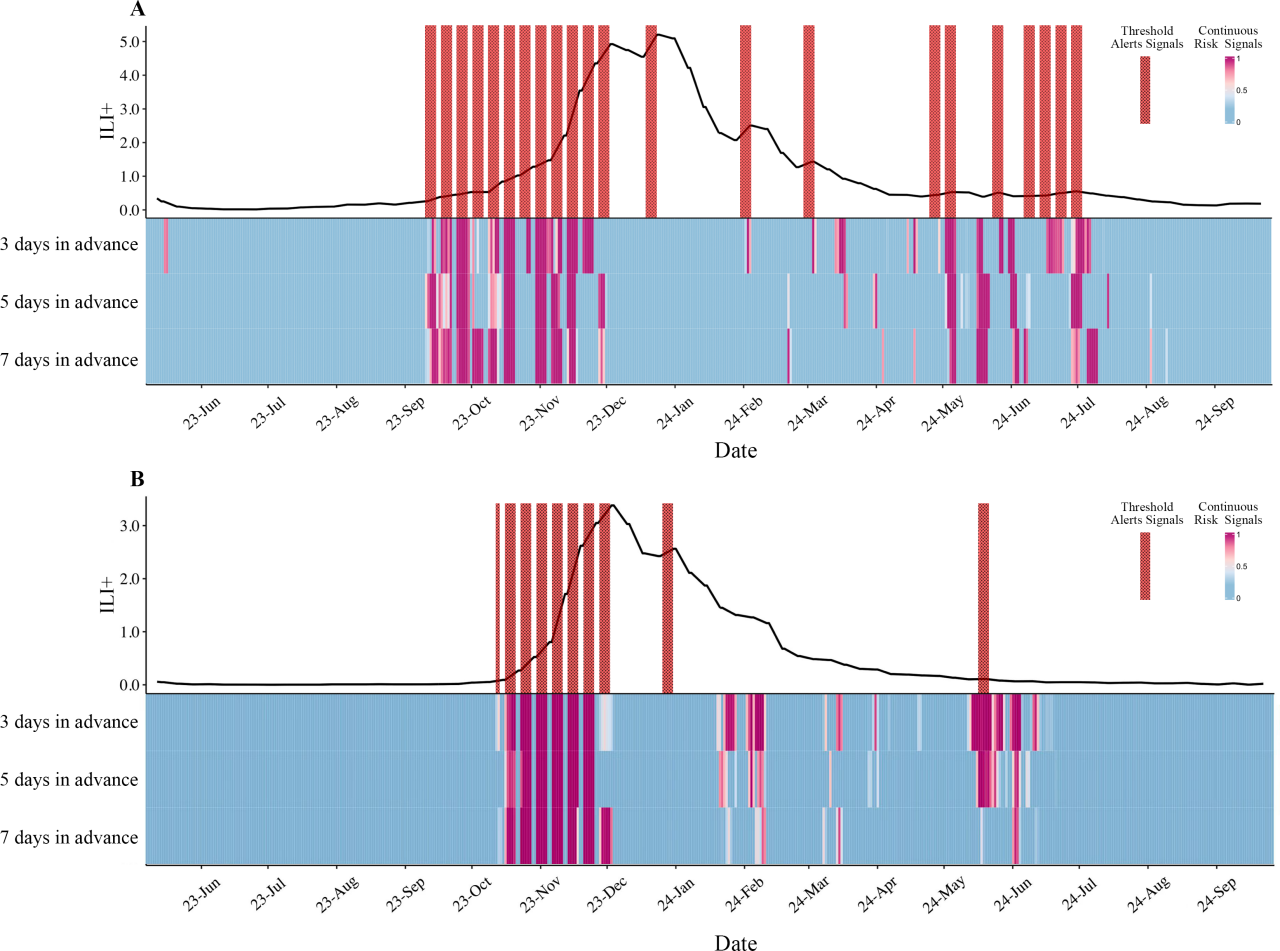
Comparison of current warning signals per 3, 5, and 7 days in advance of predicted warning signals in (A) Southern China and (B) Northern China. The black line refers to the ILI+ (%). ILI+: influenza-like illness positive rate.

Following the training and validation procedures employing the Dense ResNet model, early warning signals were issued at 3-, 5-, and 7-day intervals. The results indicated that, in Northern and Southern China, the most effective lead time is 3 days, 50 quantiles, allowing for the detection of warning signals 2 and 5 days in advance (Figure 5).

**Figure 6.**
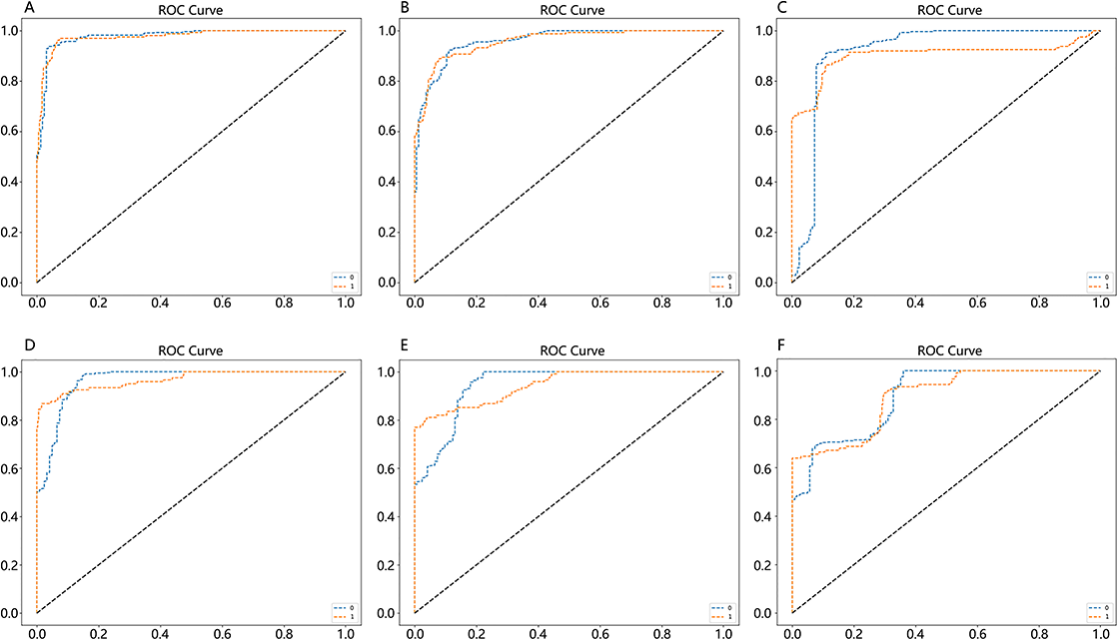
Receiver operating characteristic curves of early warning effect in Northern and Southern China. (A) Northern China at 3 days in advance; (B) Northern China at 5 days in advance; (C) Northern China at 7 days in advance; (D) Southern China at 3 days in advance; (E) Southern China at 5 days in advance; and (F) Southern China at 7 days in advance. ROC: receiver operating characteristic.

This study assessed the warning performance in Northern and Southern China, using metrics such as accuracy, recall, *F*_1_-score, and area under the curve (AUC) to validate the model. The outcomes highlight the highest AUC score for issuing warning signals 3 days in advance (Northern China: 0.94, Southern China: 0.95) (Table 1). The receiver operating characteristic curves are depicted in Figure 6.

Compared to other commonly used models (support vector machine [SVM], random forest, XGBoost [Extreme Gradient Boosting], and LSTM), the Dense ResNet model exhibits superior warning performance in Southern and Northern China, as well as in Beijing and Yunnan province (Table 2).

**Table 1.** Evaluation of early warning in Northern China and Southern China.

Days in advance and warning category		Precision	Recall	*F*_1_-score	AUC^a^ score
Northern China					
3 days					0.94
Warning 0		0.96	0.95	0.96	
Warning 1		0.89	0.93	0.91	
5 days					0.90
Warning 0		0.91	0.95	0.93	
Warning 1		0.88	0.81	0.84	
7 days					0.87
Warning 0		0.84	0.99	0.91	
Warning 1		0.99	0.60	0.75	
Southern China					
3 days					0.95
Warning 0		0.94	0.99	0.97	
Warning 1		0.98	0.81	0.89	
5 days					0.92
Warning 0		0.91	0.99	0.95	
Warning 1		0.99	0.69	0.82	
7 days					0.91
Warning 0		0.89	0.99	0.94	
Warning 1		0.99	0.61	0.76	

a AUC: area under the curve.

**Table 2.** Evaluation and comparison of the 3-day early warning results of 5 models.

Model^a^		Precision	Recall	*F*_1_-score	AUC^b^ score
Northern China					
	SVM^c^	0.87	0.67	0.76	0.81
	XGboost^d^	0.85	0.76	0.80	0.85
	RF^e^	0.79	0.75	0.77	0.85
	LSTM^f^	0.69	0.75	0.72	0.81
	Dense Resnet^g^	0.89	0.93	0.91	0.94
Southern China					
	SVM	0.89	0.80	0.84	0.89
	XGboost	0.67	0.80	0.73	0.84
	RF	0.68	0.85	0.76	0.87
	LSTM	0.76	0.66	0.70	0.87
	Dense ResNet	0.98	0.81	0.89	0.95

a The warning category for all models is 1.

b AUC: area under the curve.

c SVM: support vector machine.

d XGBoost: Extreme Gradient Boosting.

e RF: random forest.

f LSTM: long short-term memory.

g Dense ResNet: Dense Residual Network.

The results demonstrate that Dense ResNet outperforms models such as SVM, random forest, and LSTM, primarily for the following reasons. First, Dense ResNet leverages its deep network architecture, particularly through multiple dense layers and residual connections, to effectively capture complex nonlinear relationships in the data. In contrast, traditional machine learning models such as SVM and random forest, while capable of handling some degree of nonlinearity, often exhibit limitations in modeling high-dimensional data and intricate relationships. SVM typically relies on kernel functions for nonlinear mapping; however, it is less flexible compared to deep neural networks when processing large-scale data and complex features. Random forest, on the other hand, enhances the expressiveness of the model through an ensemble of decision trees. However, it reveals low performance compared to deep neural networks when dealing with highly correlated features or long-range dependencies.

Second, the Dense ResNet model benefits from hierarchical feature learning, with each dense layer progressively extracting more abstract and complex features, gradually building a representation from low-level to high-level features. In comparison, traditional models such as SVM and random forest rely more heavily on manual feature engineering or selection. While LSTM excels in processing sequential data, it depends on longer input sequences to capture complex global information and long-term dependencies, making it less efficient than Dense ResNet for learning both short-term and long-term features, as may be involved in this study.

Finally, the Dense ResNet model in this study incorporates residual connections, effectively mitigating the vanishing gradient problem and facilitating the training of deeper networks. By allowing information to skip layers, residual connections help preserve more global information, a crucial advantage when handling large-scale datasets. In contrast, models such as SVM, random forest, and LSTM lack such structural designs, which may limit their performance in modeling high-dimensional or complex data.

## Discussion

### Principal Findings

In China, seasonal influenza follows a predictable pattern, peaking during the colder months and, in some regions, during the summer. The ability to issue timely and precise warnings is crucial for public health preparedness. This study introduces a continuous probability-based warning system (0‐1 variable), moving beyond the traditional binary (0/1) warning models. This advancement significantly enhances the flexibility, accuracy, and applicability of early warning systems.

A probability-based risk assessment improves the accuracy of warning signals. Traditional early warning models [18] rely on binary classifications—either issuing a warning or not—which often leads to false alarms or missed detections. These rigid threshold-based warning systems do not account for the varying degrees of epidemic severity. In contrast, our continuous probability warning approach provides a more nuanced risk assessment, offering granular insights into the likelihood of an outbreak. This enables decision-makers to adjust intervention strategies based on the level of risk rather than adhering to a fixed binary outcome. As a result, the reliability and adaptability of early warning systems are significantly improved.

Moreover, a probability-based warning system supports tiered response strategies, which can be dynamically adjusted according to the predicted severity of an outbreak. For example, when the warning probability is moderate (0.4‐0.6), internal monitoring may be prioritized, whereas higher probabilities (≥0.7) would prompt targeted interventions such as increasing health care preparedness or reinforcing public health advisories. When the probability reaches 0.9 or above, immediate public health measures can be activated. This graduated approach prevents unnecessary disruptions in low-risk situations while ensuring timely action in high-risk scenarios.

A flexible warning system supports precise public health interventions. Another key advantage of probability-based warnings is their role in scientific policy making and public communication. Traditional threshold-based methods may trigger unnecessary alarms in low-risk years, leading to economic losses and diminishing public responsiveness to warning signals. Conversely, these methods may fail to escalate warnings quickly enough in high-risk years, resulting in delayed response measures. By shifting to a risk-probability framework, this study enables a more rational and transparent decision-making process, allowing authorities to communicate risk levels clearly to the population to prevent overreactions.

In addition, when the probability of an outbreak is low, ranging from 0.3 to 0.5, preventive measures can be targeted at high-risk groups, such as older adults and individuals with pre-existing health conditions, without imposing large-scale interventions. This precision prevention strategy minimizes unnecessary economic losses and social disruption while ensuring that those at the most significant risk receive appropriate protection.

AI-driven automation enhances the efficiency of warning responses. Beyond improving warning accuracy, continuous probability warnings pave the way for AI-driven automation in public health management. In future applications, AI-assisted decision-making systems can integrate probability forecasts into automated response frameworks. For example, when the probability is ≥0.7, automated alerts can be dispatched to health care facilities. If a probability is ≥0.9, public health advisories can be issued automatically to initiate broader containment measures. For probabilities between 0.4 and 0.6, real-time monitoring can continue internally without initiating unnecessary public concerns.

By integrating machine learning and automation, this system improves the efficiency of response, consequently reducing delays in the intervention while optimizing the allocation of health care resources. Early warning facilitates the implementation of timely interventions, leading to improved mitigation of the outbreak. The effectiveness of the proposed model was demonstrated through empirical validation. Our study shows that the machine learning model can issue warnings 5 days earlier than traditional methods, significantly improving public health preparedness [19]. Given that implementing preventive measures taken 5 days in advance can impact the infection chain by 2‐4 generations, this proactive alert system is essential for managing the dissemination of influenza. Similar findings have been observed in real-time surveillance systems for other infectious diseases, such as dengue [20], where early detection has been shown to reduce the outbreak’s severity and enhance the efficiency of implemented control measures. Moreover, our results indicate that when predicting outbreaks 3 days in advance, the model achieves the highest sensitivity, specificity, and timeliness. This suggests that while earlier warning signals provide more time for response, optimizing the trade-off between lead time and accuracy is essential. Future research should explore hybrid models that integrate transmission dynamics modeling and data-driven time-series forecasting to further improve warning precision.

Machine learning outperforms traditional threshold-based methods. Compared to the conventional fixed percentile warning system, such as the 40th percentile thresholds, our probability-based approach provides 3 key advantages, including (1) earlier detection, which is usually executed. This approach enables a crucial preparatory window for vaccine distribution, hospital readiness, and community interventions; (2) distinguishing between rising and declining trends, which often provide warning signals even after the peak of the outbreak, with probability-based warning signals dynamically adjusted based on real-time trends, unlike fixed-threshold methods; and (3) improved adaptability to different epidemic conditions, in which machine learning models learn from historical patterns, providing a flexible response mechanism even during atypical flu seasons.

A probability-based warning system enhances influenza preparedness. This study demonstrated the superior performance of probability-based machine learning models in the early warning of seasonal influenza.

### Conclusions

This study presented a novel probability-based machine learning model for the early warning of influenza, demonstrating superior accuracy, flexibility, and practical applicability. By replacing binary warning signals with probability-driven risk assessments, this approach enhances influenza preparedness and supports automated AI-driven public health responses. Future research should integrate real-time surveillance data and transmission dynamic models to improve the precision of early warning signals further. On the one hand, the Susceptible-Exposed-Infected-Recovered model can effectively depict the transmission stages and overall trends of infectious diseases among the population, providing a basic framework for understanding disease transmission. Conversely, agent-based modeling can simulate the complex interaction behaviors and heterogeneity among individuals, making the model closer to the actual communication process. In contrast, the hybrid data-driven and mechanism model provides novel prospects for predicting influenza. Data-driven models can extract potential patterns and rules from extensive historical datasets, whereas mechanism models based on understanding the principles of disease transmission provide comprehensive insights into the disease transmission process. Combining these 2 approaches mitigates the limitations of a single model and improves the accuracy and reliability of influenza prediction. Meanwhile, this hybrid model can handle scenarios of incomplete data or highly uncertain datasets, enhancing the robustness and adaptability of the model. This system enhances public health preparedness and reduces unnecessary disruptions by issuing warning signals 5 days in advance and providing a flexible, risk-adaptive approach. Compared to traditional methods, machine learning accurately distinguishes epidemic trends, minimizes false alarms, and facilitates automated AI-driven public health responses. Future research should further refine warning frameworks by integrating transmission dynamics models and real-time surveillance data, ensuring improved promptness, timeliness, and efficacy of prevention and control measures for influenza.

## Abbreviations

AI: artificial intelligence
AUC: area under the curve
ILI: influenza-like illness
ILI+: influenza-like illness positive rate
LSTM: long short-term memory
SVM: support vector machine
XGBoost: Extreme Gradient Boosting
